# Clinical characteristics and risk factors associated with consultation‐confirmed delirium in hospitalized patients

**DOI:** 10.1002/pcn5.70385

**Published:** 2026-07-30

**Authors:** Megumi Matsuda, Kazuhiko Yamamuro, Masatsugu Ikeuchi, Akihiro Minami, Izumi Harada, Takahira Yamauchi, Tetsuro Kitamura, Yasuyo Kobayashi, Yukako Ishida, Yusuke Inagaki, Jyunko Minamiguchi, Akira Kido, Takashi Okada

**Affiliations:** ^1^ Psychiatric Center Nara Medical University Hospital Kashihara Japan; ^2^ Center for Health Control Nara Medical University Kashihara Japan; ^3^ Department of Psychiatry Nara Medical University Kashihara Japan; ^4^ Department of Neurosurgery Nara Medical University Hospital Kashihara Japan; ^5^ Department of Rehabilitation Medicine Nara Medical University Kashihara Japan; ^6^ Department of Nursing Nara Medical University Hospital Kashihara Japan; ^7^ Department of Community‐Based Rehabilitation Systems Science Nara Medical University Kashihara Japan

**Keywords:** consultation‐liaison psychiatry, delirium, dementia, hospitalization, risk factors

## Abstract

**Aim:**

Delirium, a serious neuropsychiatric hospitalization complication, is a manifestation of underlying neurocognitive vulnerability rather than an acute event. In Japan, a nationwide medical policy mandates early hospital‐wide delirium risk screening to evaluate predefined risk factors in inpatient populations. We aimed to identify demographic and clinical factors independently associated with referral‐based, consultation‐confirmed delirium.

**Methods:**

We conducted a retrospective observational study of consecutive hospitalized patients at Nara Medical University Hospital between June 2022 and November 2023, without exclusion criteria. Delirium risk factors were assessed within 3 days of admission using a standardized checklist based on national criteria. Delirium was diagnosed following specialist consultation according to the International Classification of Diseases, 10th Revision (ICD‐10), and the Diagnostic and Statistical Manual of Mental Disorders, Fifth Edition, Text Revision criteria. Logistic regression analyses were performed to identify independent association with consultation‐confirmed delirium.

**Results:**

Among 20,980 patients (mean age, 67.1 ± 17.0 years; males, 55.0%), 209 (1.0%) were diagnosed with delirium following specialist consultation. Overall, 56.4% met at least one predefined risk criterion at admission. In multivariable analyses adjusted for age and sex, dementia (odds ratio [OR], 3.995; 95% confidence interval [CI], 2.647–6.029), history of delirium (OR, 3.649; 95% CI, 2.158–6.170), and heavy alcohol use (OR, 1.648; 95% CI, 1.002–2.710) were independently associated with consultation‐confirmed delirium.

**Conclusions:**

In this large cohort evaluated under a nationwide screening framework, consultation‐confirmed delirium was independently associated with baseline neurocognitive vulnerability (dementia and history of delirium) and heavy alcohol use, rather than chronological age alone, supporting vulnerability‐focused risk stratification in routine clinical practice.

## INTRODUCTION

Delirium is an acute neuropsychiatric syndrome characterized by disturbances in attention, awareness, and cognition that develop over a short period and fluctuate in severity.^1^ As one of the most frequent and serious complications of hospitalization, it occurs particularly among older adults and medically vulnerable patients. Delirium is associated with adverse clinical outcomes, including prolonged hospitalization and increased mortality.^2^, ^3^, ^4^, ^5^ Increasing evidence indicates that delirium is not merely a transient condition but a risk factor for subsequent dementia and persistent cognitive deterioration.^5^, ^6^

The reported incidence of delirium varies according to clinical settings. In general medical wards, delirium affects approximately 10%–30% of hospitalized adults, increasing to 30%–50% among older patients, and exceeding 50% in high‐risk settings, such as intensive care units and postoperative populations.^7^, ^8^, ^9^, ^10^ However, these estimates are largely derived from systematic screening studies. In routine clinical practice, delirium—particularly the hypoactive form—remains under‐recognized.^8^, ^9^ Consequently, cases identified through specialist consultation may represent only a subset of delirium presentations rather than its full spectrum. Given the aging population, early identification and prevention of delirium remain important clinical priorities. Delirium is widely conceptualized as resulting from interactions between predisposing vulnerabilities and precipitating insults.^11^ Established predisposing risk factors include advanced age, dementia, prior delirium, structural brain disease, and chronic alcohol use.^12^, ^13^, ^14^, ^15^ Among these, dementia is one of the strongest predictors, conferring a three‐ to five‐fold increased risk during acute illness.^10^, ^13^ Precipitating factors include surgery, high‐risk medications, infection, metabolic disturbances, and environmental stressors.^14^, ^15^, ^16^ This multifactorial model underscores the importance of early risk stratification and raises the question of whether chronological age independently contributes to delirium risk beyond baseline vulnerability.

Although delirium management has traditionally required specialist involvement, increasing awareness and guideline dissemination have enabled non‐psychiatric physicians to initiate early recognition and first‐line management.^17^, ^18^ Multicomponent nonpharmacological interventions implemented by general medical teams reduce delirium incidence and severity.^17^, ^19^ Therefore, referrals to psychiatric or dementia care teams may represent only a subset of delirium cases within consultation‐based systems. Despite extensive research, several limitations remain. Many studies have focused on selected populations, such as intensive care unit patients or specific surgical cohorts, limiting generalizability.^8^, ^9^, ^20^ In addition, heterogeneity in screening practices and diagnostic pathways complicates comparisons across institutions, and standardized hospital‐wide risk assessment has not been uniformly implemented.^19^

In Japan, a national reimbursement framework for delirium prevention mandates early risk assessment using predefined criteria within 3 days of admission, followed by implementation of preventive interventions.^21^ This nationwide approach provides an opportunity to examine the distribution of predefined delirium risk factors in an unselected hospital population and evaluate their association with consultation‐confirmed delirium in a real‐world setting. Because this framework applies to all hospitalized patients without exclusion criteria, it enables analysis across the full clinical spectrum of inpatient care. However, data on the distribution of predefined risk factors among consecutive hospitalized patients and their association with consultation‐confirmed delirium remain limited.^21^ Clarifying this relationship may improve the understanding of how predefined risk screening relates to referral‐based diagnoses and inform hospital‐wide delirium prevention strategies.

Therefore, this study was aimed at describing the demographic and clinical characteristics of a large consecutive cohort of hospitalized patients without exclusion criteria; examining the distribution of predefined delirium risk factors based on the Japanese national screening framework; and identifying demographic and clinical factors independently associated with referral and consultation‐confirmed delirium within a consultation‐based system using logistic regression analyses in a real‐world hospital setting, focusing on the relative contributions of chronological age and baseline vulnerability factors.

## METHODS

### Study design and participants

This retrospective observational study was conducted at Nara Medical University Hospital. Medical records of all consecutive patients hospitalized between June 2022 and November 2023 were reviewed. All hospitalized patients during the study period were included, and no exclusion criteria were applied. In total, 20,980 patients were included in the analysis. Demographic information, consultation status, diagnosis of delirium, and predefined clinical risk factors were extracted from electronic medical records. No additional interventions were performed as part of this study. Patients in the “no consultation” group were those for whom no consultation with the dementia care team or psychiatry department was requested during hospitalization.

### Definition of delirium risk factors

Delirium risk factors were defined according to the Japanese national medical fee revision framework for delirium prevention established by the Ministry of Health, Labour and Welfare (2020). Under this framework, hospitalized patients are required to undergo early risk assessment to identify those at high risk for delirium and initiate appropriate preventive interventions. Risk factor screening was systematically conducted within 3 days of hospital admission using a standardized institutional checklist based on national criteria. The checklist was completed as part of routine inpatient care and documented in the electronic medical record system.

Predefined risk factors included age ≥70 years, organic brain disorder, dementia, heavy alcohol use, history of delirium, use of medications associated with increased delirium risk, and surgery under general anesthesia (including elective and emergency procedures). Heavy alcohol use was defined as documented habitual alcohol consumption requiring clinical attention according to the institutional screening checklist, including history of alcohol dependence, withdrawal symptoms, or physician‐documented excessive intake. Medications associated with delirium risk included benzodiazepines, anticholinergic agents, opioids, corticosteroids, and sedative‐hypnotics, as specified in the institutional screening checklist. Organic brain disorder included prior stroke, traumatic brain injury, brain tumor, Parkinsonian disorders, and other structural brain diseases documented in the medical record. Patients in the no‐consultation group were those for whom no consultation with psychiatry or the dementia care team was requested during hospitalization. These patients might have included individuals with no suspected delirium as well as patients managed by primary teams without specialist referral.

Patients with no identified risk factors during the initial screening period were classified as “none applicable.”

### Diagnosis of delirium

When delirium was suspected, the primary clinical physician requested consultation with the dementia care team, the psychiatry department, or both. The consulting team established the diagnosis of delirium based on clinical assessment according to the International Classification of Diseases, 10th Revision (ICD‐10), and the Diagnostic and Statistical Manual of Mental Disorders, Fifth Edition, Text Revision (DSM‐5‐TR). Following confirmation of the diagnosis, appropriate management was initiated. All diagnoses were documented in the electronic medical record system as part of routine clinical practice.

### Ethics approval

This study was conducted in accordance with the Declaration of Helsinki and was approved by the Ethics Committee of Nara Medical University (approval number: 3993). Given the retrospective study design and use of anonymized clinical data, the requirement for written informed consent was waived by the Ethics Committee. Instead, an opt‐out procedure was implemented, and study information was publicly disclosed to provide patients with the opportunity to decline participation.

### Statistical analyses

All statistical analyses were performed using IBM SPSS Statistics for Windows (version 26.0; IBM Corp.). Continuous variables are presented as mean ± standard deviation (SD), and categorical variables are presented as numbers (percentage).

Comparisons among the three groups (consultation without delirium, consultation with delirium, and no consultation) were conducted using one‐way analysis of variance (ANOVA) for continuous variables and chi‐square (*χ* ^2^) tests for categorical variables. Age‐stratified analyses were conducted using *χ* ^2^ tests to evaluate differences across age categories.

Logistic regression analyses were conducted to identify the factors associated with consultation‐confirmed delirium within a consultation‐based clinical system. Crude odds ratios (ORs) and 95% confidence intervals (CIs) were estimated using unadjusted models. Multivariable logistic regression models were also constructed: Model 1 was adjusted for age and Model 2 was adjusted for age and sex. Crude and adjusted ORs with 95% CIs are reported. All statistical tests were two‐sided, and a *p*‐value < 0.05 was considered statistically significant.

## RESULTS

### Baseline demographics and clinical characteristics

A total of 20,980 hospitalized patients were included in the analysis (Table 1). The mean age was 67.09 years (SD = 16.96), and 55.0% of the patients were males. In‐hospital consultations with the dementia care team or psychiatry department were conducted for 509 patients (2.4%). The distribution of consultation departments among patients with and without delirium is presented in Supporting Information S1: Tables S1 and S2, respectively. The primary clinical reasons for psychiatric consultation are summarized in Supporting Information S1: Table S3. Delirium was diagnosed in 209 patients (1.0%). Regarding predefined delirium risk factors, 11,823 patients (56.4%) were aged ≥70 years; organic brain disorders, dementia, heavy alcohol use, and history of delirium were present in 1243 (5.9%), 697 (3.3%), 1302 (6.2%), and 314 (1.5%) patients, respectively. Medications associated with increased delirium risk were used by 1830 (8.7%) patients, and 5251 (25.3%) patients underwent surgery under general anesthesia. A total of 4446 patients (21.2%) had no identified delirium risk factors at baseline.

**Table 1:** Demographic and clinical characteristics of the study participants.

Variable	Value
Age, years, mean (SD)	67.09 (16.96)
Sex, males, *n* (%)	11,541 (55.01)
Consultation with the dementia care team or the psychiatry department, *n* (%)	509 (2.43)
Diagnosis of delirium	209 (1.00)
Risk factors for delirium, *n* (%)	
Age ≥70 years	11,823 (56.36)
Organic brain disorder	1243 (5.93)
Dementia	697 (3.32)
Heavy alcohol use	1302 (6.21)
History of delirium	314 (1.50)
Use of medications associated with increased risk	1830 (8.72)
Surgery under general anesthesia	5251 (25.30)
None applicable	4446 (21.20)

*Note*: Data are presented as means (standard deviations [SD]) for continuous variables and as numbers (percentages) for categorical variables. Percentages were calculated based on the total study population (*N* = 20,980). Delirium risk factors were predefined according to the study protocol and included age ≥70 years, organic brain disorder, dementia, heavy alcohol use, a history of delirium, use of medications associated with increased delirium risk, and surgery under general anesthesia, including both elective and emergency procedures. “None applicable” indicates participants without any identified delirium risk factors at baseline. Consultation refers to an in‐hospital consultation with the dementia care team or psychiatric department.

### Comparison according to consultation status and delirium diagnosis

Demographic and clinical characteristics were compared among three patient groups (Table 2): patients in the consultation group without delirium (*N* = 300), patients in the consultation group diagnosed with delirium (*N* = 209), and patients in the no‐consultation group (*N* = 20,470). Age showed a significant difference across the groups (*p* < 0.001), with the highest mean age observed in patients diagnosed with delirium in the consultation group (71.85 ± 11.63 years). The proportion of patients aged ≥70 years was also significantly higher in this group (74.6%) than that in the consultation group without delirium (34.0%) and the no‐consultation group (56.5%; *p* < 0.001). Dementia and history of delirium were significantly more prevalent among patients diagnosed with delirium (both *p* < 0.001). Heavy alcohol use was more frequent in the consultation groups (*p* < 0.01). In contrast, the prevalence of organic brain disorders and sex distribution did not significantly differ among the groups. Interestingly, surgery under general anesthesia was less frequent in both consultation groups than that in the non‐consultation group (*p* < 0.001). The absence of any identified risk factors was less common in the consultation group with delirium (9.6%) than that in the other groups (*p* < 0.001).

**Table 2:** Comparison of demographic and clinical characteristics according to consultation status and delirium diagnosis.

	Consultation group		No‐consultation group	
Variable	No delirium	Diagnosis of delirium		*p*‐value or *χ* ^2^
	*N* = 300	*N* = 209	*N* = 20,470	
Age, year, mean (SD)	60.93 (17.84)	71.85 (11.63)	67.16 (16.90)	**<0.001**
Sex, male, *n* (%)	154 (51.33)	123 (58.85)	11,263 (55.02)	0.24
Risk factors for delirium, *n* (%)				
Age ≥70 years	102 (34.00)	**156** (**74.64)**	11,565 (56.50)	**<0.001**
Organic brain disorder	22 (7.33)	**18** (**8.61)**	1203 (5.88)	0.15
Dementia	26 (8.67)	**35** (**16.75)**	636 (3.11)	**<0.001**
Heavy alcohol use	**29** (**9.67)**	19 (9.09)	1255 (6.13)	**<0.01**
History of delirium	13 (4.33)	**18** (**8.61)**	283 (1.38)	**<0.001**
Use of medications associated with increased risk	**93** (**31.00)**	24 (11.48)	1713 (8.37)	**<0.001**
Surgery under general anesthesia	44 (14.67)	36 (17.22)	**5171** (**25.26)**	**<0.001**
None applicable	**68** (**22.67)**	20 (9.57)	4359 (21.29)	**<0.001**

*Note*: Demographic and clinical characteristics were compared among three groups: patients in the consultation group without delirium (*N* = 300), patients in the consultation group diagnosed with delirium (*N* = 209), and patients in the no‐consultation group (*N* = 20,470). Data are presented as means (standard deviations [SD]) for continuous variables and as numbers (percentages) for categorical variables. Age was compared using one‐way analysis of variance (ANOVA), and categorical variables were compared using *χ* ^2^ tests. For categorical variables, *χ* ^2^ statistics and corresponding *p*‐values are reported. *p*‐values indicate overall group differences. Delirium risk factors included age ≥70 years, organic brain disorder, dementia, heavy alcohol use, a history of delirium, use of medications associated with increased delirium risk, and surgery under general anesthesia, including both elective and emergency procedures. For categorical variables, percentages presented in bold indicate the highest value among the three groups, highlighting the group with the greatest prevalence for each characteristic. Statistical significance was defined as a two‐sided *p* < 0.05.

### Age‐stratified distribution of delirium and associated risk factors

Age‐stratified analyses revealed significant differences across all age categories (Table 3). The prevalence of delirium increased progressively with age, from 0% in patients aged <40 years to 1.57% in those aged >80 years (*p* < 0.001). Dementia and history of delirium were markedly more prevalent in the older age groups, particularly among patients aged ≥70 years (*p* < 0.001). In contrast, heavy alcohol use was more prevalent in the middle‐aged groups and declined in the oldest age category (*p* < 0.001). The proportion of patients undergoing surgery under general anesthesia decreased progressively with age, whereas that of patients with no identified risk factors increased up to the 60–69‐year age group and was absent in patients aged ≥70 years, reflecting the predefined age‐based risk criterion.

**Table 3:** Age‐stratified distribution of demographic characteristics, consultation status, delirium diagnosis, and associated risk factors.

Age, years	<20	≥20 and <30	≥30 and <40	≥40 and <50	≥50 and <60	≥60 and <70	≥70 and <80	>80	
	*N* = 482	*N* = 479	*N* = 649	*N* = 1395	*N* = 2336	*N* = 3815	*N* = 7166	*N* = 4657	*χ* ^2^
Sex, males, *n* (%)	278 (57.68)	226 (47.18)	266 (40.99)	689 (49.39)	1,156 (49.49)	2,264 (59.34)	**4,333** (**60.46)**	2417 (51.90)	**<0.001**
Consultation with the dementia care team or the psychiatry department, *n* (%)	13 (2.70)	30 (6.26)	13 (2.00)	54 (3.87)	65 (2.78)	76 (1.99)	141 (1.97)	**117** (**2.51)**	**<0.001**
Diagnosis of delirium	0 (0)	0 (0)	0 (0)	8 (0.57)	18 (0.77)	27 (0.71)	83 (1.16)	**73** (**1.57)**	**<0.001**
Risk factors for delirium, *n* (%)									
Age ≥70 years	0 (0)	0 (0)	0 (0)	0 (0)	0 (0)	0 (0)	**7166** (**100)**	**4657** (**100)**	**<0.001**
Organic brain disorder	19 (3.94)	18 (3.76)	24 (3.70)	81 (5.81)	159 (6.81)	226 (5.92)	382 (5.33)	**334** (**7.17)**	**<0.001**
Dementia	0 (0)	0 (0)	0 (0)	2 (0.14)	2 (0.09)	26 (0.68)	187 (2.61)	**480** (**10.31)**	**<0.001**
Heavy alcohol use	0 (0)	19 (3.97)	47 (7.24)	**156** (**11.18)**	225 (9.63)	313 (8.20)	400 (5.58)	142 (3.05)	**<0.001**
History of delirium	0 (0)	2 (0.42)	4 (0.62)	6 (0.43)	22 (0.94)	46 (1.21)	109 (1.52)	**125** (**2.68)**	**<0.001**
Use of medications associated with increased risk	38 (7.88)	48 (10.02)	86 (13.25)	190 (13.62)	**320** (**13.70)**	405 (10.62)	440 (6.14)	303 (6.51)	**<0.001**
Surgery under general anesthesia	**265** (**54.98)**	193 (40.29)	276 (42.53)	542 (38.88)	707 (30.27)	989 (25.93)	1552 (21.66)	727 (15.61)	**<0.001**
None applicable	176 (36.51)	230 (48.02)	274 (42.22)	588 (42.15)	1121 (47.99)	**2057** (**53.92)**	0 (0)	0 (0)	**<0.001**

*Note*: This table summarizes demographic characteristics, consultation status, diagnosis of delirium, and delirium risk factors stratified by age group (<20 [*N* = 482], ≥20–<30 [*N* = 479], ≥30–<40 [*N* = 649], ≥40–<50 [*N* = 1395], ≥50–<60 [*N* = 2336], ≥60–<70 [*N* = 3815], ≥70–<80 [*N* = 7166], and >80 years [*N* = 4657]). Data are presented as numbers (percentages) for each age group. Differences across the age groups were evaluated using chi‐square (*χ* ^2^) test. Reported *p*‐values indicate the overall comparisons among all age categories. Delirium risk factors included age ≥70 years, organic brain disorder, dementia, heavy alcohol use, history of delirium, use of medications associated with increased delirium risk, and surgery under general anesthesia, including both elective and emergency procedures. For each variable, the highest percentage across age groups is presented in bold font. Statistical significance was defined as a two‐sided *p*‐value < 0.05.

### Logistic regression analyses of factors associated with consultation‐confirmed delirium

Logistic regression analyses were performed to examine the associations of age and sex with delirium (Table 4).

**Table 4:** Logistic regression analysis of factors associated with delirium.

Variables	Unadjusted OR [95% CI]	*p*‐value
Sex	1.475 [1.110–1.961]	7.45 × 10^−3^
Age	1.032 [1.020–1.043]	2.74 × 10^−8^

*Note*: Odds ratios (ORs) and 95% confidence intervals (CIs) were estimated using logistic regression analysis. Sex was coded as male = 1 and female = 2. Age was considered a continuous variable. Unadjusted ORs represent crude associations with delirium. Statistical significance was defined as *p* < 0.05.

In the unadjusted analyses, increasing age was significantly associated with delirium (OR = 1.032 per year; 95% CI, 1.020–1.043; *p* < 0.001). Female sex was also significantly associated with a higher likelihood of delirium compared with male sex (OR = 1.475; 95% CI, 1.110–1.961; *p* = 0.007).

Association between predefined clinical risk factors and delirium was evaluated using unadjusted and multivariable logistic regression models (Table 5). In the unadjusted analyses, dementia (OR = 4.028; 95% CI, 2.702–6.005; *p* < 0.001), history of delirium (OR = 3.872; 95% CI, 2.293–6.540, *p* < 0.001), heavy alcohol use (OR = 1.720; 95% CI, 1.048–2.824; *p* = 0.032), and age ≥70 years (OR = 1.653; 95% CI, 1.074–2.543; *p* = 0.020) were significantly associated with delirium. In contrast, organic brain disorders, use of medications associated with increased risk, surgery under general anesthesia, and absence of identified risk factors did not exhibit significant associations. After adjustment for age (Model 1), dementia, history of delirium, and heavy alcohol use remained significantly associated with delirium. In the fully adjusted model controlling for age and sex (Model 2), dementia (OR = 3.995; 95% CI, 2.647–6.029; *p* < 0.001), history of delirium (OR = 3.649; 95% CI, 2.158–6.170; *p* < 0.001), and heavy alcohol use (OR = 1.648; 95% CI, 1.002–2.710; *p* = 0.049) remained independently associated with delirium.

**Table 5:** Unadjusted and multivariable logistic regression analyses of delirium risk factors.

Variables	Unadjusted OR [95% CI]	*p*‐value	^1^Adjusted OR [95% CI]	*p*‐value	^2^Adjusted OR [95% CI]	*p*‐value
Age ≥70 years	**1.653 [1.074–2.543]**	**0.02**	1.091 [0.612–1.945]	0.77	1.034 [0.582–1.838]	0.91
Organic brain disorder	1.136 [0.688–1.876]	0.62	1.119 [0.677–1.848]	0.66	1.099 [0.665–1.817]	0.71
Dementia	**4.028 [2.702–6.005]**	**7.91 × 10** ^**−12**^	**3.685 [2.450–5.544]**	**3.82 × 10** ^**−10**^	**3.995 [2.647–6.029]**	**4.20 × 10** ^**−11**^
Heavy alcohol use	**1.720 [1.048–2.824]**	**3.18 × 10** ^**−2**^	**1.724 [1.049–2.835]**	**3.17 × 10** ^**−2**^	**1.648 [1.002–2.710]**	**4.91 × 10** ^**−2**^
History of delirium	**3.872 [2.293–6.540]**	**4.10 × 10** ^**−7**^	**3.764 [2.229–6.358]**	**7.17 × 10** ^**−7**^	**3.649 [2.158–6.170]**	**1.37 × 10** ^**−6**^
Use of medications associated with increased risk	1.460 [0.926–2.303]	0.62	1.464 [0.929–2.309]	0.10	1.538 [0.976–2.425]	0.06
Surgery under general anesthesia	0.689 [0.467–1.016]	0.06	0.716 [0.485–1.057]	0.09	0.732 [0.497–1.080]	0.67
None applicable	0.708 [0.377–1.330]	0.28	0.700 [0.373–1.313]	0.27	0.672 [0.358–1.260]	0.67

*Note*: Odds ratios (ORs) and 95% confidence intervals (CIs) were estimated using logistic regression analysis. Unadjusted ORs represent crude associations. Model 1 was adjusted for age. Model 2 was adjusted for age and sex. Delirium risk factors were predefined according to the study protocol and included age ≥70 years, organic brain disorder, dementia, heavy alcohol use, history of delirium, use of medications associated with increased delirium risk, and surgery under general anesthesia (including both elective and emergency procedures). “None applicable” indicates participants without any identified delirium risk factors at baseline. Statistical significance was defined as *p* < 0.05.

After multivariable adjustment, age ≥70 years, organic brain disorder, use of medications associated with increased risk, surgery under general anesthesia, and absence of predefined risk factors were not independently associated with delirium.

## DISCUSSION

In this large retrospective cohort of 20,980 consecutive hospitalized patients, we examined predefined delirium risk factors within a national screening framework and identified factors associated with consultation‐confirmed delirium. Three main findings emerged from this study. First, more than half of patients met at least one predefined risk criterion, whereas consultation‐confirmed delirium was diagnosed in only 1.0%. Second, dementia, history of delirium, and heavy alcohol use were independently associated with referral and consultation‐confirmed delirium. Third, age ≥70 years and surgery under general anesthesia were not independently associated with delirium after adjustment for age and sex. These findings reflect how predefined screening criteria relate to referral‐based diagnoses in routine clinical practice. Importantly, because delirium was ascertained through specialist consultation rather than systematic bedside screening, the identified risk factors should be interpreted as determinants of specialist referral and consultation‐confirmed diagnosis rather than as a reflection of the true delirium epidemiology in the broader inpatient population.

The overall prevalence of delirium (1.0%) in this study was lower than that reported in systematic screening‐based studies.^22^, ^23^ This discrepancy likely reflects the consultation‐based diagnostic pathway used at our institution. The low prevalence of consultation‐confirmed delirium likely reflects the consultation‐based diagnostic pathway. Mild or transient cases managed by primary teams without specialist referral may not have been captured. Therefore, the present findings should be interpreted as identifying factors associated with referral and specialist‐confirmed delirium rather than the overall occurrence of delirium. Delirium, particularly the hypoactive subtype, is frequently under‐recognized in routine clinical settings.^24^, ^25^, ^26^ Consultation‐confirmed delirium may therefore represent a subset of cases identified through specialist referral.^27^ Accordingly, the present findings should be interpreted as factors associated with referral and consultation‐confirmed delirium rather than the overall occurrence of delirium. The consultation‐based diagnostic pathway may have introduced a differential detection bias related to delirium motor subtypes. Hyperactive delirium, characterized by agitation, combativeness, and psychomotor excitation, is more readily observed by primary clinical teams and is more likely to prompt specialist referral compared with the hypoactive subtype, which manifests as reduced responsiveness and psychomotor slowing and is frequently unrecognized or misattributed to fatigue or depression.^24^, ^25^ Patients with dementia may be particularly prone to presenting with hyperactive or mixed delirium phenotypes during acute illness, as disrupted cholinergic and dopaminergic signaling associated with neurodegenerative pathology may lower the threshold for agitated behavioral responses to physiological stressors.^28^ This could partly explain the strong association between dementia and consultation‐confirmed delirium observed in the present study. Similarly, alcohol withdrawal‐related delirium characteristically manifests with hyperactive features, including autonomic arousal and agitation, which are highly visible and typically require urgent specialist involvement.^29^, ^30^ In contrast, patients without these baseline vulnerabilities who develop hypoactive delirium may be managed by primary teams or remain undiagnosed, which results in their underrepresentation in the consultation‐confirmed group. Consequently, the identified risk factors—dementia, history of delirium, and heavy alcohol use—may reflect not only genuine predisposing vulnerability but also a referral bias toward conditions that are more commonly associated with the hyperactive phenotype. This limitation should be considered when generalizing the findings of the present study to a broader spectrum of delirium presentations.

Advanced age has consistently been identified as a major risk factor for delirium.^31^, ^32^ In our unadjusted analyses, increasing age was associated with delirium; however, age ≥70 years was not independently associated after accounting for dementia and prior delirium. This suggests that chronological age may reflect underlying vulnerability rather than an independent determinant. However, this interpretation warrants caution. Dementia and prior delirium, which were included as covariates, may function as age‐related mediators rather than independent confounders, raising the possibility of overadjustment that could attenuate the estimated association of chronological age with delirium. Moreover, information on important confounders, including frailty, baseline functional impairment, illness severity, ICU admission, and polypharmacy, were unavailable for adjustment, and residual confounding cannot be excluded. Previous studies indicate that frailty, cognitive impairment, and structural brain disease contribute to age‐related risk.^33^, ^34^ These findings are consistent with a vulnerability model in which baseline susceptibility accounts for much of the association between aging and delirium, although the independent contribution of chronological age cannot be definitively excluded given the limitations of the current analyses. Dementia was the strongest independent predictor in our cohort, conferring approximately a fourfold increase in odds after multivariable adjustment. This finding is consistent with evidence demonstrating a bidirectional relationship between delirium and dementia.^6^ Neurodegenerative processes may reduce cognitive reserve and increase susceptibility to acute physiological stressors through neuroinflammatory and network‐level mechanisms.^28^ These findings underscore the importance of proactive delirium prevention strategies in patients with pre‐existing cognitive impairment.

A history of delirium independently predicted subsequent delirium, supporting evidence that recurrence reflects persistent vulnerability.^6^, ^18^ Structural and functional neural alterations after an initial episode may lower the threshold for future episodes during subsequent medical stress.^18^ Incorporating prior delirium history into early risk stratification may improve preventive targeting. Heavy alcohol use was independently associated with delirium. Alcohol‐related neurotoxicity, withdrawal phenomena, and metabolic dysregulation contribute to delirium pathophysiology.^16^, ^28^ Because alcohol use is often under‐recognized in acute care settings, systematic assessment may be important in prevention frameworks. In contrast, surgery under general anesthesia was not independently associated with delirium after multivariable adjustment. Although postoperative delirium is common,^35^, ^36^ its occurrence is influenced by predisposing factors such as dementia and frailty.^37^ Surgical exposure may therefore function primarily as a precipitating factor rather than an independent determinant of delirium risk. More than half of hospitalized patients met at least one predefined risk criterion under the national framework, indicating widespread vulnerability in the inpatient population. Standardized early risk identification may facilitate targeted preventive strategies.^38^, ^39^ However, the relatively low rate of consultation‐confirmed delirium suggests that predefined risk does not uniformly translate into diagnosed cases within consultation‐based systems. This finding highlights the distinction between vulnerability screening and referral‐based diagnosis.

The distribution of referral departments reflects real‐world consultation patterns rather than the incidence of delirium within each specialty. Several limitations should be considered. First, delirium diagnoses were consultation‐based and the prevalence was low (1.0%), suggesting under‐recognition of mild or hypoactive cases. Accordingly, the findings reflect factors associated with referral and consultation‐confirmed delirium rather than overall delirium occurrence. Second, standardized bedside screening tools, such as the Confusion Assessment Method (CAM), CAM‐ICU, or 4AT, were not uniformly implemented across clinical settings. The absence of such tools reduces diagnostic sensitivity and reproducibility, likely contributing to the low observed prevalence of delirium (1.0%) relative to that reported in systematic screening‐based studies. Accordingly, the risk factor estimates reported here reflect the association with referral and consultation‐confirmed delirium rather than delirium as ascertained using validated bedside instruments, and may not be directly comparable with estimates from screening‐based cohorts. Furthermore, delirium subtypes were not systematically classified. The absence of systematic subtype classification precludes assessment of whether the identified risk factors differentially predict hyperactive versus hypoactive delirium, and whether the consultation‐based pathway selectively captured patients with more behaviorally prominent presentations. Third, this was a single‐center study at a tertiary hospital, potentially limiting generalizability. Fourth, several important confounding variables known to influence the delirium risk, including frailty, illness severity scores, ICU admission, presence of infection or sepsis, baseline functional impairment, and polypharmacy, were unavailable for adjustment. The inability to adjust for these factors limits the validity of the multivariable risk estimates and means that residual confounding cannot be excluded. Fifth, several exposure variables were defined broadly and heterogeneously. Pharmacologically distinct medication classes—benzodiazepines, anticholinergic agents, opioids, corticosteroids, and sedative‐hypnotics—were grouped together as a single binary variable representing “medications associated with delirium risk,” without accounting for individual drug class, dosage, duration, timing of exposure, or cumulative medication burden. Similarly, “surgery under general anesthesia” was treated as a binary variable despite substantial heterogeneity in postoperative delirium risk across different surgical procedures, anesthetic approaches, and perioperative clinical contexts. This level of variable granularity may limit the interpretability and specificity of the risk estimates for these exposures. Finally, treatment strategies and response to treatment were not quantitatively evaluated because of the retrospective design of this study.

In conclusion, in a large cohort of consecutive hospitalized patients evaluated within a standardized national screening framework, referral and consultation‐confirmed delirium were independently associated with baseline neurocognitive vulnerability and heavy alcohol use within a consultation‐based clinical pathway, whereas chronological age and surgical exposure were not independent predictors after multivariable adjustment. These findings should be interpreted within the context of a consultation‐based ascertainment design, which may not fully capture the true spectrum of delirium in the inpatient population. Nevertheless, the results support vulnerability‐focused early risk stratification as a practical approach in routine hospital care.

## AUTHOR CONTRIBUTIONS

Megumi Matsuda and Kazuhiko Yamamuro contributed equally to this study. Megumi Matsuda and Kazuhiko Yamamuro designed the study. Megumi Matsuda, Kazuhiko Yamamuro, Masatsugu Ikeuchi, Akihiro Minami, Izumi Harada, Takahira Yamauchi, Tetsuro Kitamura, Yasuyo Kobayashi, Yukako Ishida, Yusuke Inagaki, Jyunko Minamiguchi, Akira Kido, and Takashi Okada collected the data. Kazuhiko Yamamuro performed the statistical analyses. Megumi Matsuda, Kazuhiko Yamamuro, Masatsugu Ikeuchi, Izumi Harada, Takahira Yamauchi, Tetsuro Kitamura, Yasuyo Kobayashi, Yukako Ishida, Yusuke Inagaki, Jyunko Minamiguchi, Akira Kido, and Takashi Okada contributed to data interpretation. Megumi Matsuda and Kazuhiko Yamamuro drafted the manuscript. All the authors critically revised and approved the final manuscript version.

## CONFLICT OF INTEREST STATEMENT

The authors declare no conflicts of interest.

## ETHICS APPROVAL STATEMENT

This study was conducted in accordance with the Declaration of Helsinki and was approved by the Ethics Committee of Nara Medical University (approval number: 3993).

## PATIENT CONSENT STATEMENT

The requirement for written informed consent was waived by the Ethics Committee of Nara Medical University owing to the retrospective study design and use of anonymized clinical data. An opt‐out procedure was implemented, and the study information was publicly disclosed to provide patients the opportunity to decline participation.

## CLINICAL TRIAL REGISTRATION

Not applicable. This was a retrospective observational study; thus, it was not registered as a clinical trial.

## Supporting information

Supporting File 1

## Data Availability

The data that support the findings of this study are available on request from the corresponding author. The data are not publicly available due to privacy or ethical restrictions.
