# Supplementary material for: Clinical characteristics and risk factors associated with consultation‐confirmed delirium in hospitalized patients
Source: PCN Rep. 2026 Jul 30;5(3):e70385. doi: 10.1002/pcn5.70385 (PMC13420208; doi:10.1002/pcn5.70385)
Supplement: Supplementary file 1 — Supporting File 1 [file PCN5-5-e70385-s001.docx]

**Table S1. Distribution of consultation departments for patients diagnosed with delirium**

| Department | n |
| --- | --- |
| **Cardiology** | 31 |
| **Gastrointestinal Surgery** | 23 |
| **Gastroenterology** | 20 |
| **Respiratory Medicine** | 19 |
| **Dermatology** | 14 |
| **Nephrology** | 11 |
| **Neurology** | 9 |
| **Emergency Medicine** | 9 |
| **Neurosurgery** | 9 |
| **Cardiovascular Surgery** | 8 |
| **General Medicine** | 7 |
| **Urology** | 7 |
| **Radiology** | 6 |
| **Plastic Surgery** | 6 |
| **Otorhinolaryngology (ENT)** | 4 |
| **Orthopaedic Surgery** | 3 |
| **Infectious Diseases** | 2 |
| **Endocrinology and Diabetology** | 2 |
| **Oral and Maxillofacial Surgery** | 2 |
| **Medical Oncology** | 1 |
| **Haematology** | 1 |
| **Gynaecology** | 1 |
| **Obstetrics and Gynaecology** | 1 |
| **Anaesthesiology** | 1 |

This table summarises the distribution of the primary consultation departments for patients diagnosed with delirium. The values indicate the number of consultations in each department during the study period. The departments are presented in descending order of frequency.

**Table S2. Distribution of consultation departments for patients without delirium diagnosis**

| Department | n |
| --- | --- |
| **Emergency Medicine** | 119 |
| **Gastroenterology** | 21 |
| **Neurology** | 18 |
| **Respiratory Medicine** | 15 |
| **Infectious Diseases** | 12 |
| **Endocrinology and Diabetology** | 12 |
| **Urology** | 11 |
| **General Medicine** | 10 |
| **Gynaecology** | 10 |
| **Cardiology** | 9 |
| **Nephrology** | 9 |
| **Gastrointestinal Surgery** | 9 |
| **Orthopaedic Surgery** | 9 |
| **Neurosurgery** | 7 |
| **Haematology** | 4 |
| **Cardiovascular Surgery** | 3 |
| **Obstetrics and Gynaecology** | 3 |
| **Oral and Maxillofacial Surgery** | 2 |
| **Otorhinolaryngology (ENT)** | 2 |
| **Dermatology** | 2 |
| **Radiology** | 1 |
| **Plastic Surgery** | 1 |

The table summarises the distribution of the clinical departments that requested consultations for patients who were evaluated but did not meet the delirium diagnostic criteria. The values represent the total number of consultations in each department during the study period. The departments are presented in descending order of frequency.

**Table S3. Reasons for psychiatric consultation**

| Reasons for Consultation | n |
| --- | --- |
| **Post–Suicide Attempt Evaluation** | 95 |
| **Assessment of Psychiatric Symptoms** | 93 |
| **Co-management of Psychiatric Disorders** | 65 |
| Cognitive and Dementia Assessment (including BPSD) | 45 |
| **Neurocognitive Function Assessment** | 2 |

The table presents the distribution of consultation requests according to primary clinical indications. Each patient was classified according to the primary reason for referral. The values represent the total number of cases in each category during the study period.
